# Viscosity, Conductivity, and Electrochemical Property of Dicyanamide Ionic Liquids

**DOI:** 10.3389/fchem.2018.00059

**Published:** 2018-03-15

**Authors:** Wen-Li Yuan, Xiao Yang, Ling He, Ying Xue, Song Qin, Guo-Hong Tao

**Affiliations:** College of Chemistry, Sichuan University, Chengdu, China

**Keywords:** dicyanamide ionic liquids, electrochemistry, viscosity, Walden plots, diffusion coefficients

## Abstract

The instructive structure-property relationships of ionic liquids (ILs) can be put to task-specific design of new functionalized ILs. The dicyanamide (DCA) ILs are typical CHN type ILs which are halogen free, chemical stable, low-viscous, and fuel-rich. The transport properties of DCA ionic liquids are significant for their applications as solvents, electrolytes, and hypergolic propellants. This work systematically investigates several important transport properties of four DCA ILs ([C_4_mim][N(CN)_2_], [C_4_m_2_im][N(CN)_2_], N_4442_[N(CN)_2_], and N_8444_[N(CN)_2_]) including viscosity, conductivity, and electrochemical property at different temperatures. The melting points, temperature-dependent viscosities and conductivities reveal the structure-activity relationship of four DCA ILs. From the Walden plots, the imidazolium cations exhibit stronger cation–anion attraction than the ammonium cations. DCA ILs have relatively high values of electrochemical windows (EWs), which indicates that the DCA ILs are potential candidates for electrolytes in electrochemical applications. The cyclic voltammograms of Eu(III) in these DCA ILs at GC working electrode at various temperatures 303–333 K consists of quasi-reversible waves. The electrochemical properties of the DCA ILs are also dominated by the cationic structures. The current intensity (*i*_p_), the diffusion coefficients (*D*_o_), the charge transfer rate constants (*k*_s_) of Eu(III) in DCA ILs all increased with the molar conductivities increased. The cationic structure-transport property relationships of DCA ILs were constructed for designing novel functionalized ILs to fulfill specific demands.

## Introduction

Ionic liquids (ILs) have many desirable properties to serve as soft functional materials including solvents (Rogers and Seddon, 2003), catalysts (Hallett and Welton, 2011), lubricants (Fan et al., 2014), electrolytes (Armand et al., 2009), extractants (Wieszczycka et al., 2013), absorbents (Brennecke and Gurkan, 2010), magnetic fluids (Nacham et al., 2015), optical fluids (He et al., 2015; Zhao et al., 2015), and propellants (Tao et al., 2008; He et al., 2010; Gao et al., 2015; Yin et al., 2016). In comparison with traditional molecular solvents, ILs have many unique physical properties such as negligible vapor pressure, large liquidus range, high thermal stability, and wide electrochemical window (Galinski et al., 2006; Andriyko et al., 2009). Functionalized ILs/task-specific ILs have already become general pattern to prepare new ionic liquid materials based on the remarkable “design” capacity of ILs (Muller et al., 2013). The design processes of novel materials sorely depend on empirical rules. Therefore, researchers are always on the lookout for the instructive structure-property relationships of ILs that can be put to task-specific design of new functionalized ILs.

Dicyanamide (DCA) ILs are good nonaqueous solvents of transition metal salts because of the ligand ability of DCA anion as a Lewis base (Simons et al., 2014). Most of the metal chlorides are insoluble in tetrafluoroborate, hexafluorophosphate, and bis(trifluoromethylsulfonyl)imide ILs, but well dissolved into DCA ILs due to the high complexing ability of DCA anion (Schmeissera and Eldik, 2014). Furthermore, the structure of DCA anion is much easier to be oxidize by fuming nitric acid, which can be used as new hypergolic propellants. DCA ILs possess lower viscosity than most of common ILs such as tetrafluoroborate, hexafluorophosphate, and bis(trifluoromethylsulfonyl)imide counterparts (MacFarlane et al., 2002). Lower viscosity implies higher conductivity and more efficient mass transport for the applications of electrochemical and rocket bipropellant system (Yoshida et al., 2007). Meanwhile, the CHN component of DCA anion gives these ILs an inbuilt advantage over other halogen-containing ILs to the facility and environment. These features would highly benefit the electrochemical studies. Transport properties are very important for electrochemical solvents. However, in fact, some DCA ILs may be a little viscous. Then their physicochemical properties would be much different with the conventional DCA ILs. The structure-property relationships of DCA ILs, especially the effects of cationic structures on the transport properties including viscosity, conductivity, and electrochemical properties, are still not clear enough.

**Scheme 1:**
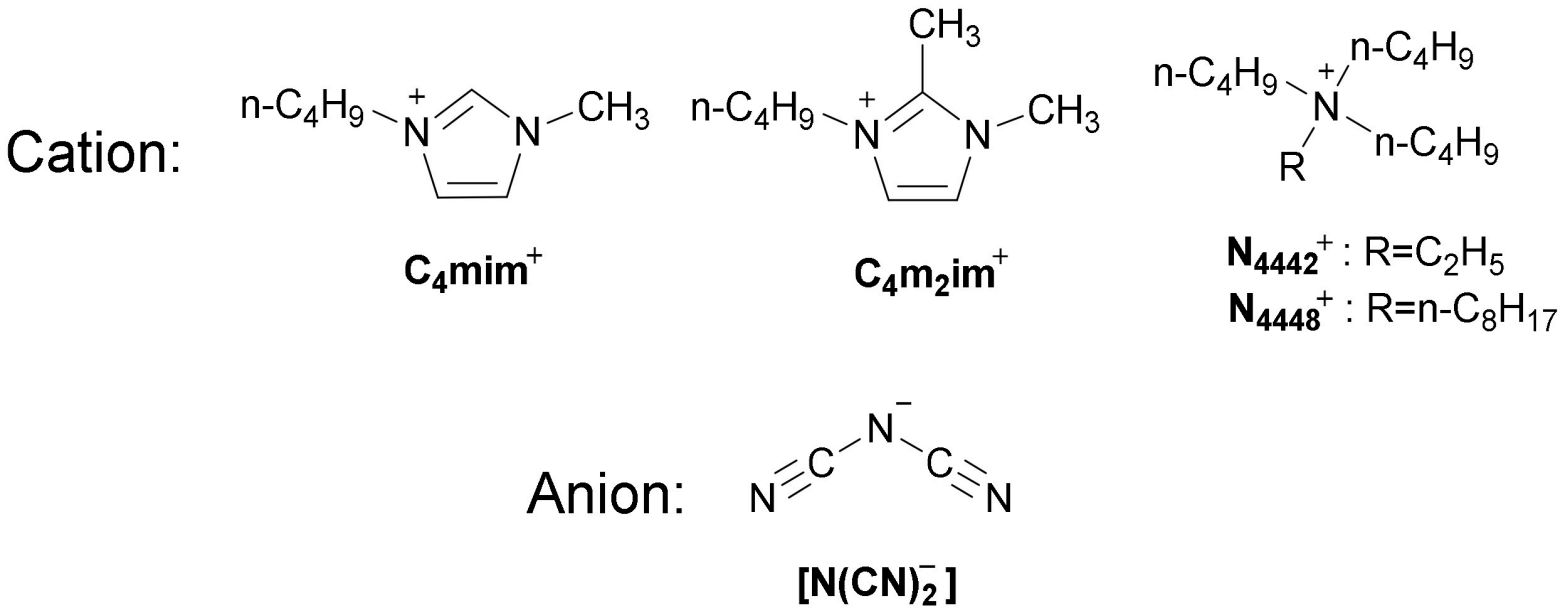
Molecular structures of the DCA ILs.

Herein, a series of DCA ILs was designed and synthesized to find the structure-activity relationship of different cation structures, including 1-butyl-3-methylimidazolium dicyanamide ([C_4_mim][N(CN)_2_]), 1-butyl-2,3-dimethylimidazolium dicyanamide ([C_4_m_2_im][N(CN)_2_]), N-ethyl-N,N,N-tributylammonium dicyanamide (N_4442_[N(CN)_2_]), and N-octyl-N,N,N-tributylammonium dicyanamide (N_8444_[N(CN)_2_]), (Scheme 1). Besides the basic characterization, the electrochemical behaviors of Eu(III) in DCA ILs were also investigated by cyclic voltammetry method. The diffusion coefficients, charge transfer rate constants, formal potentials and Gibbs energy were estimated based on cyclic voltammetry curves, from which we can find the effects of cation structures on the transport properties in DCA ILs.

## Materials and methods

All chemicals were commercially available with analytical grade. 1-Butyl-3-methylimidazolium bromide ([C_4_mim]Br), 1-butyl-2,3-dimethylimidazolium bromide ([C_4_m_2_im]Br), N-ethyl-N,N,N-tributylammonium bromide (N_4442_Br), and N-octyl-N,N,N-tributylammonium bromide (N_8444_Br) were synthesized by the Menschutkin reaction of corresponding imidazole or N,N,N-tributylamine with the appropriate alkyl halides according to the literature method (Gordon, 2002). Silver dicyanamide (Ag[N(CN)_2_]) was prepared by the metathesis of Na[N(CN)_2_] with AgNO_3_ in the dark in distilled water.

### [C_4_mim][N(CN)_2_]

[C_4_mim]Br (4.39 g, 20 mmol) were dissolved in 50 mL distilled water, and then Ag[N(CN)_2_] (3.68 g, 21 mmol) was added. The resulting suspension was stirred overnight in the dark at room temperature. The byproduct AgBr along with the unreacted Ag[N(CN)_2_] were removed by filtration. The filtrate was collected and dried under vacuum at 373 K to yield [C_4_mim][N(CN)_2_] as a colorless liquid. Yield: 3.96 g (96%). IR (KBr, cm^−1^): 3148 (w), 3102 (w), 2962 (m), 2937 (w), 2873 (w), 2232 (s), 2194 (s), 2132 (vs.), 1570 (s), 1464 (s), 1380 (w), 1310 (s), 1169 (s), 1113 (w), 1024 (w), 948 (w), 903 (w), 846 (w), 754 (m), 652 (m), 622 (s). ^1^H-NMR (DMSO-d_6_, δ/ppm): 0.89 (t, 3H, *J* = 7.2 Hz), 1.27 (m, 2H), 1.77 (m, 2H), 3.85 (s, 3H), 4.16 (t, 2H, *J* = 7.2 Hz), 7.68 (s, 1H), 7.75 (s, 1H), 9.11 (s, 1H). ^13^C-NMR (DMSO-d_6_, δ/ppm): 13.12, 18.68, 31.25, 35.63, 48.44, 122.13, 123.48, 136.41. Anal. Calcd for C_10_H_15_N_5_ (205.26): C, 58.51; H, 7.37; N, 34.12; found: C, 58.32; H, 7.56; N, 33.97.

### [C_4_m_2_im][N(CN)_2_]

A similar procedure was followed as that described for [C_4_mim][N(CN)_2_]. [C_4_m_2_im]Br (4.66 g, 20 mmol) and Ag[N(CN)_2_] (3.68 g, 21 mmol) were reacted in 50 mL distilled water to obtain a light yellow liquid. Yield: 4.03 g (92%). IR (KBr, cm^−1^): 3178 (vw), 3134 (w), 3016 (vw), 2963 (m), 2937 (w), 2229 (s), 2192 (s), 2132 (vs.), 1588 (s), 1538 (s), 1417 (s), 1251 (w), 1185 (m), 1134 (m), 1044 (w), 903 (m), 828 (w), 755 (s), 665 (s), 626 (w). ^1^H-NMR (DMSO-d_6_, δ/ppm): 0.89 (t, 3H, *J* = 7.2 Hz), 1.29 (m, 2H), 1.69 (m, 2H), 2.60 (s, 3H), 3.77 (s, 3H), 4.12 (t, 2H, *J* = 7.2 Hz), 7.63 (s, 1H), 7.66 (s, 1H). ^13^C-NMR (DMSO-d_6_, δ/ppm): 9.14, 13.24, 18.77, 31.07, 34.58, 47.23, 120.71, 122.14, 144.06. Anal. Calcd for C_11_H_17_N_5_ (219.29): C, 60.25; H, 7.81; N, 31.94; found: C, 60.13; H, 7.88; N, 31.84.

### N_4442_[N(CN)_2_]

A similar procedure was followed as that described for [C_4_mim][N(CN)_2_]. N_4442_Br (5.87 g, 20 mmol) and Ag[N(CN)_2_] (3.68 g, 21 mmol) were reacted in 50 mL distilled water to obtain a yellow liquid. Yield: 4.76 g (85%). IR (KBr, cm^−1^): 3134 (w), 2964 (s), 2933 (s), 2875 (s), 2226 (s), 2190 (s), 2130 (vs.), 1638 (w), 1568 (w), 1467 (s), 1386 (m), 1306 (s), 1158 (w), 1096 (w), 1062 (w), 1032 (w), 898 (w), 804 (w), 739 (w). ^1^H-NMR (DMSO-d_6_, δ/ppm): 0.93 (t, 3H, *J* = 7.2 Hz), 1.18 (t, 3H, *J* = 7.2 Hz), 1.32 (m, 2H), 1.58 (m, 2H), 3.18 (s, 2H), 3.29 (s, 2H). ^13^C-NMR (DMSO-d_6_, δ/ppm): 7.03, 13.16, 18.95, 22.79, 56.79, 118.85. Anal. Calcd for C_16_H_32_N_4_ (280.45): C, 68.52; H, 11.50; N, 19.98; found: C, 68.74; H, 11.58; N, 19.62.

### N_8444_[N(CN)_2_]

A similar procedure was followed as that described for [C_4_mim][N(CN)_2_]. N_8444_Br (7.54 g, 20 mmol) and Ag[N(CN)_2_] (3.68 g, 21 mmol) were reacted in 50 mL distilled water to obtain a yellow liquid. Yield: 6.47 g (89%). IR (KBr, cm^−1^): 3132 (w), 2961 (s), 2930 (s), 2872 (s), 2225 (s), 2189 (s), 2130 (vs.), 1637 (w), 1569 (w), 1466 (m), 1381 (w), 1306 (m), 1152 (w), 1110 (w), 1067 (w), 1032 (w), 895 (w), 799 (w), 740 (w). ^1^H-NMR (DMSO-d_6_, δ/ppm): 0.87 (t, 3H, *J* = 7.2 Hz), 0.94 (t, 3H, *J* = 7.2 Hz), 1.31 (m, 2H), 1.58 (m, 2H), 3.01 (s, 2H), 3.20 (s, 2H). ^13^C-NMR (DMSO-d_6_, δ/ppm): 13.27, 19.08, 20.85, 21.94, 22.96, 25.11, 25.62, 28.30, 31.01, 51.78, 57.47, 118.97. Anal. Calcd for C_22_H_44_N_4_ (364.61): C, 72.47; H, 12.16; N, 15.37; found: C, 72.36; H, 12.45; N, 15.14.

### Measurements methods

Infrared spectra (IR) were recorded using KBr plates on a Bruker ALPHA-ATR spectrophotometer. ^1^H and ^13^C NMR spectra were recorded on a Bruker AVANCE III HD nuclear magnetic resonance spectrometer with DMSO-d_6_ as locking solvent. ^1^H and ^13^C chemical shifts are reported in ppm from TMS with the solvent resonance as the internal standard (DMSO, δ = 2.50). Thermogravimetric analysis (TGA) measurements were accomplished on a NETZSCH TG 209F1 instrument by heating samples at 10 K min^−1^ from 298 to 873 K in a dynamic nitrogen atmosphere at flow rate of 70 mL min^−1^. Differential scanning calorimetry (DSC) measurements were performed on a TA Q20 calorimeter equipped with a cool accessory and calibrated with pure indium. Measurements were performed at a heating rate of 10 K min^−1^ in sealed aluminum pans with a nitrogen flow rate of 20 mL min^−1^. Elemental analyses (C, H, N) were performed on an Flash 1112 Series EA elemental analyzer. Densities were measured by pycnometer method. Viscosities were measured with a NDJ-1B-1 viscometer. Conductivity measurements were recorded on a DDSJ-308A conductivity meter with a Q/YXLG133 conductance electrode. Cyclic voltammetry measurements were carried out in a standard three-electrode electrochemical cell with a platinum rod counter electrode and a silver/silver ion (0.1 M Ag^+^ in CH_3_CN) acted as the quasi-reference electrode. The working electrode was a glassy carbon (GC) rod with the area of 0.1256 cm^2^. Experiments were took from 303 to 333K under the nitrogen atmosphere. The electrochemical cell had a single leak-tight compartment and all the electrodes were placed in the same compartment. The ionic liquid solutions were prepared by dissolving EuCl_3_ (anhydrous, 99.9% Eu, purchased from Jiangxi Xinzheng Chemicals) into the ILs, and dried in vacuum for 12 h at 373 K.

## Results and discussion

### Thermal behaviors

The glass transition temperatures (*T*_g_), crystallization temperatures (*T*_c_), melting points (*T*_m_), and decomposition temperatures (*T*_d_) of four DCA ILs are summarized in Table 1. These DCA ILs are thermally stable, with *T*_d_ over 500 K. The structural differences in their cations could give the changes in their decomposition temperatures. Imidazolium cation could yield DCA ILs with higher thermal stability than quaternary ammonium cation. Furthermore, the highest decomposition temperature was found from [C_4_m_2_im][N(CN)_2_] than [C_4_mim][N(CN)_2_]. The reason for the increased thermal stability is most likely the presence of methyl group relative to active hydrogen attached to the C(2) position of imidazolium framework.

**Table 1:** Thermal properties of DCA ILs: [C_4_mim][N(CN)_2_], [C_4_m_2_im][N(CN)_2_], N_4442_[N(CN)_2_], and N_8444_[N(CN)_2_].

**Ionic liquid**	***T*_g_ (K)**	***T*_c_ (K)**	***T*_m_ (K)**	***T*_d_ (K)**
[C_4_mim][N(CN)_2_]	178	–	–	546
[C_4_m_2_im][N(CN)_2_]	192	243	299	573
N_4442_[N(CN)_2_]	203	–	–	510
N_8444_[N(CN)_2_]	206	270	–	531

### Viscosity

**Figure 1:**
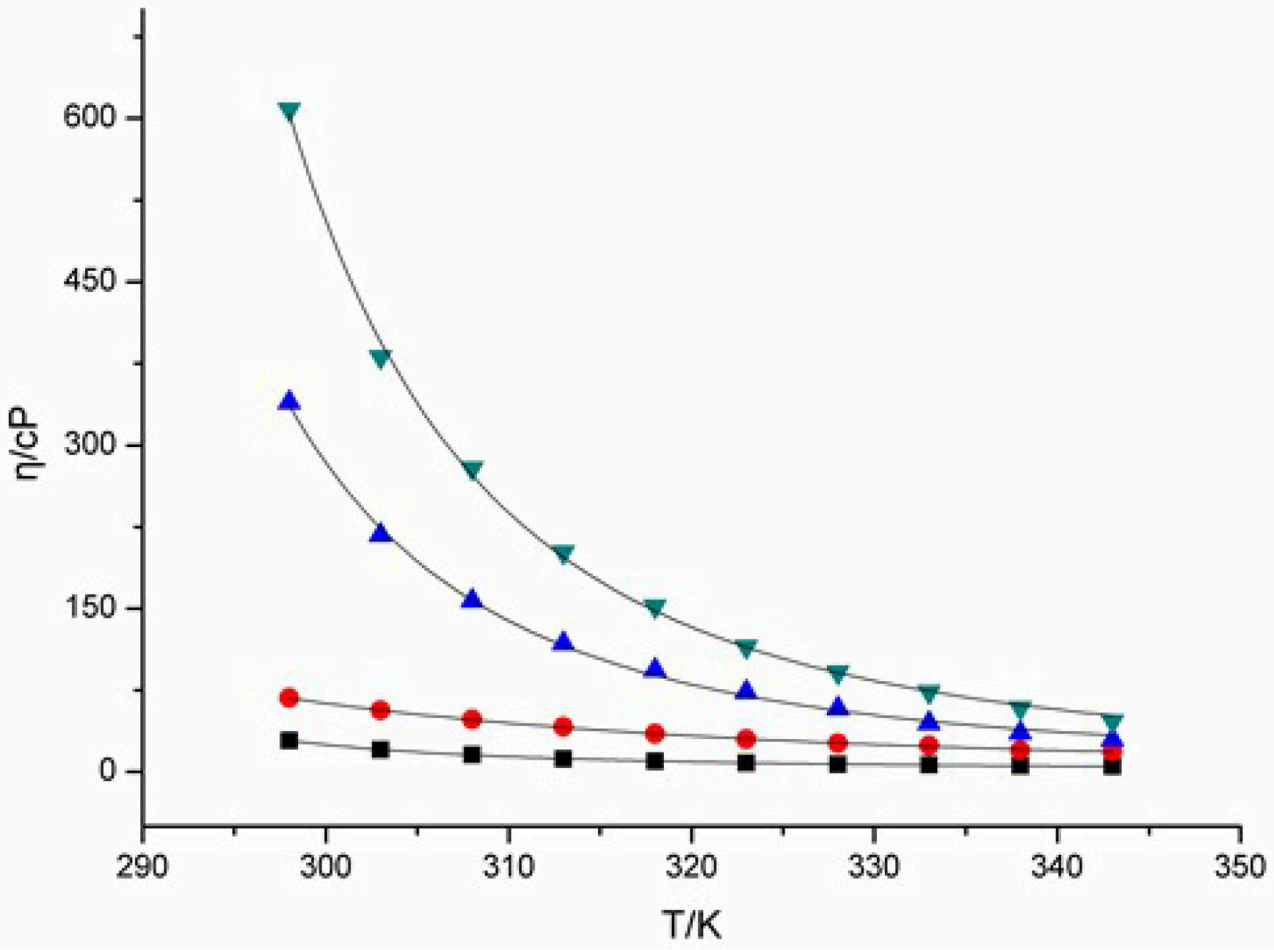
Viscosities (η) of [C_4_mim][N(CN)_2_] (black), [C_4_m_2_im][N(CN)_2_] (red), N_4442_[N(CN)_2_] (blue), and N_8444_[N(CN)_2_] (green) at different temperatures.

Viscosity of ILs is a key feature associated with their liquid characteristic, and thus clearly affects their charge transport capacity. Change in viscosity of ILs can give intimate changes in their transport properties, including conductivity, diffusion coefficient, and charge transfer rate etc. The viscosities of four DCA ILs were recorded at different temperatures and summarized in Figure 1. The combination of 1-butyl-3-methylimidazolium cation with DCA anion yields room-temperature IL [C_4_mim][N(CN)_2_] with viscosity as low as 29 cP at 298 K. The viscosity of [C_4_m_2_im][N(CN)_2_], with a methyl group attached to C2, increases to 68 cP. While, N_4442_[N(CN)_2_] and N_8444_[N(CN)_2_] with quaternary ammonium cation produce DCA ILs in higher viscosity. The viscosity values shows the salts with imidazolium cations are less viscous than the quaternary ammonium-based ILs. Asymmetric N-subsitituted imidazolium cations have been noted to be suitable for the design of low-viscous, room-temperature ILs. In these cases, the synergistic effect of the charge delocalization and planarity leads to low viscous ILs.

Hydrogen bonding is an important factor affected the viscosity of ILs. Fewer hydrogen bonds may lead to lower viscosity of IL (Kowsari et al., 2014). However, the DCA IL [C_4_mim][N(CN)_2_] with active hydrogen on C(2) exhibits lower viscosity than [C_4_m_2_im][N(CN)_2_]. For the quaternary ammonium DCA ILs, less hydrogen bonds in N_4442_[N(CN)_2_] and N_8444_[N(CN)_2_] are not give descent in their viscosity, relative to the imidazolium DCA ILs. The C(2) methyl of [C_4_m_2_im][N(CN)_2_] may increase the van der Waals interactions and decrease the entropy, leading to its viscosity bigger than that of C_4_mim[N(CN)_2_]. Meanwhile, the lengthening of alkyl chain also increases the van der Waals interactions, resulting in that N_8444_[N(CN)_2_] is more viscous than N_4442_[N(CN)_2_]. On the other hand, the imidazolium cation contains a conjugated cation structure. The positive charge of imidazolium cation is well distributed, which remarkably weakens the Coulomb interactions among ions. As a result, the viscosity of imidazolium DCA ILs are lower than that of quaternary ammonium DCA ILs. Therefore, the cationic structures may influence the viscosity of ILs through the synergistic effect of hydrogen-bond, van der Waals (vdW) attractive force, entropy and charge distribution.

Normally, the viscosity and the melting point have positive correlations. Low melting ILs should have lower viscosity and better fluidity (He et al., 2009). The DCA ILs with similar structure such as [C_4_m_2_im][N(CN)_2_] and [C_4_mim][N(CN)_2_] are in well accord with this feature. However, no positive correlation of viscosity vs. melting point was found for the DCA ILs with different cationic frameworks.

The relation between the viscosity and temperature for [C_4_m_2_im][N(CN)_2_], [C_4_m_2_im][N(CN)_2_], N_4442_[N(CN)_2_], and N_8444_[N(CN)_2_] is described in Figure 1. A rapid decrease in the viscosities is found in the DCA ILs as the temperature increases. A glassy or supercooled feature of the DCA ILs was observed. A rapid increase in the viscosity and a slowing descent of the structural relaxation occurs, on approaching the glass transition temperature. Such feature is also noted in other ILs (Tao et al., 2012). This temperature dependence of the viscosity η can be well described by the Vogel-Fulcher-Tammann (VFT) empirical equation suitable for glass-forming liquids (Equation 1; He et al., 2011; Smith et al., 2013):

(1) $$\begin{array}{r} \eta \left(T\right)=\eta_{0}exp\left(\frac{DT_{0}}{T-T_{0}}\right) \end{array}$$

where η is the viscosity, *T* is the temperature, *T*_0_ corresponds to the characteristic temperature at which η is infinite, η_0_ is a reference viscosity, and *D* is a constant presenting the structural “strength” of the system. The VFT fit curves in the equation are also shown in Figure 1. The viscosity vs. temperature graphs can be fit well to the VFT model, with a fit *R*^2^ > 0.999. The *T*_0_ values of [C_4_mim][N(CN)_2_], [C_4_m_2_im][N(CN)_2_], N_4442_[N(CN)_2_] and N_8444_[N(CN)_2_] ILs are estimated to be 249, 136, 123, and 187 K, respectively. The Arrhenius plot of the viscosity vs. temperature was also fitted. However, lower *R*^2^ > 0.99 was obtained. This plot of viscosity indicates that these DCA ILs display no-Arrhenius temperature behavior.

From Figure 1, a rapid decrease in the viscosities is found in the DCA ILs as the temperature increases. The influence of temperature is very significant on the viscosity at lower temperature. At a higher temperature 343 K, the viscosity of [C_4_mim][N(CN)_2_] is only 6.1 cP and that of [C_4_m_2_im][N(CN)_2_] is 18.8 cP, which are lower than that of N_4442_[N(CN)_2_] (29.6 cP) and N_8444_[N(CN)_2_] (46.4 cP). The strength of the momentum transfer of quaternary ammonium DCA ILs is more temperature-dependent than imidazolium DCA ILs.

### Conductivity

Conductivity of ILs originates from the inherent motion of cations and anions in ILs under electric potential difference, which is of great importance as an electrolyte for electrochemical application (Jin et al., 2008). The cationic structure has a remarkable influence on the conductivities of the four DCA ILs. At 298 K, the value of conductivity for [C_4_mim][N(CN)_2_] reaches 10.09 mS cm^−1^, which is comparable to the best non-aqueous solvent/electrolyte systems (Hapiot and Lagrost, 2008). A reduce in conductivity is found for [C_4_m_2_im][N(CN)_2_] (2.88 mS cm^−1^). Compared with the imidazolium DCA ILs, N_4442_[N(CN)_2_] and N_8444_[N(CN)_2_] exhibit much less conductivities, with the values of 50.8 μS cm^−1^ and 10.77 μS cm^−1^, respectively. The four DCA ILs have similar electrical charge, and would be all expected to possess high conductivities because they are composed of entirely of ions. However, the hundreds times difference of the conductivities indicates the available charge carries is not the only factor to high conductivities. Because of the ion aggregation/pairing, the large ion size could cause the reduction of ion mobility and then the reduction of available charge carries. Although low conductivity is not a general expectative property for ILs, it is still very interesting for studying the structure-property relationships. The conductivity of the quaternary ammonium DCA ILs are lower than other common room temperature ILs (Hapiot and Lagrost, 2008). It is predictable that N_8444_[N(CN)_2_] is not the one that owns the lowest conductivity in DCA ILs. The lower one may be expected if a quaternary ammonium with larger ion size is introduced.

**Figure 2:**
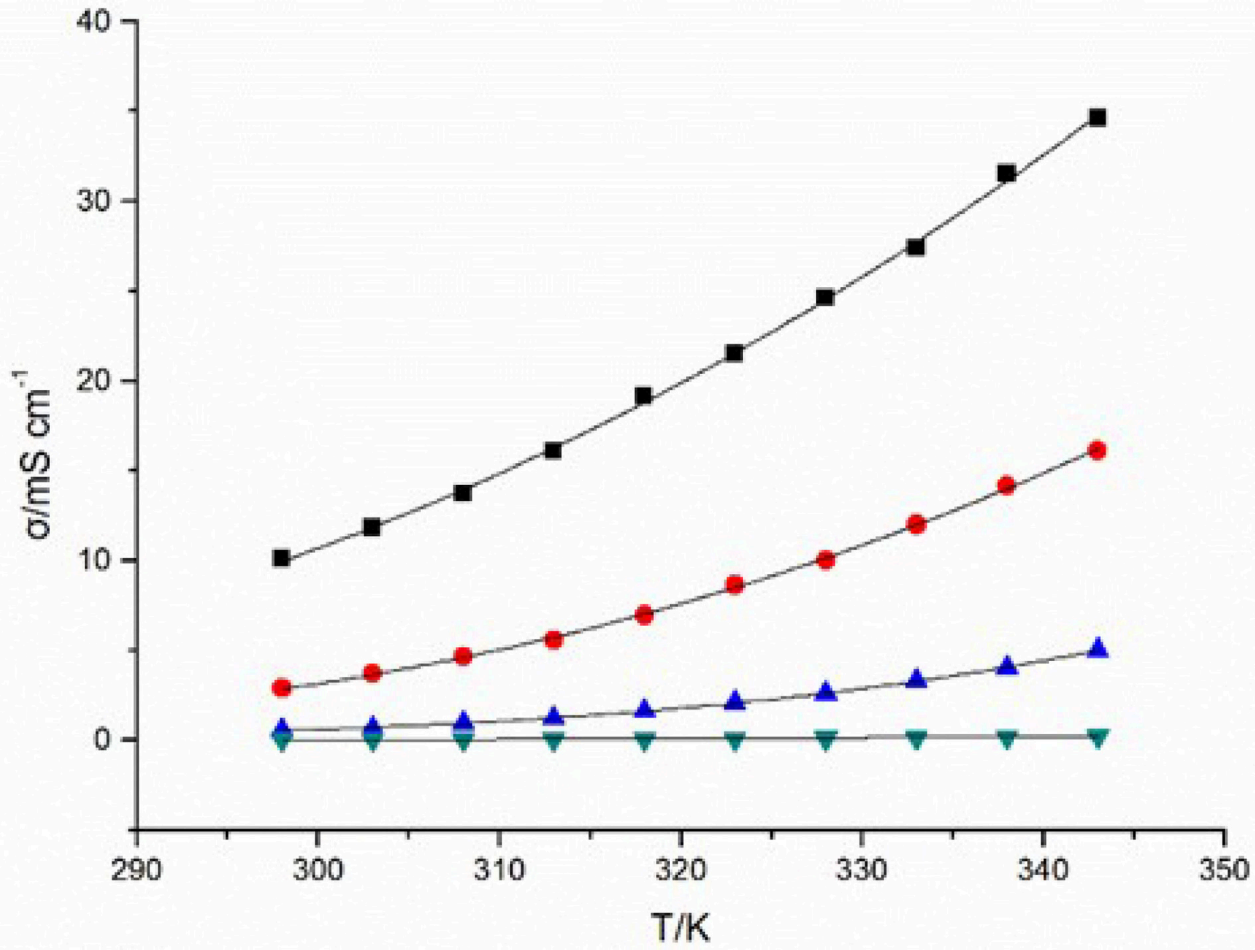
Conductivities (σ) of [C_4_mim][N(CN)_2_] (black), [C_4_m_2_im][N(CN)_2_] (red), N_4442_[N(CN)_2_] (blue), and N_8444_[N(CN)_2_] (green) at different temperatures.

The temperature and the viscosity of ILs inevitably affect the conductivity of ILs. The plots of temperature-dependent conductivity for the DCA ILs are shown in Figure 2. These curves of temperature dependence the conductivity (σ) also can be fit well by the VFT equation with the variance R^2^ > 0.999. (Equation 2):

(2) $$\begin{array}{r} \sigma \;=\;\sigma_{0\;}exp\left(\frac{DT_{0}}{T-T_{0}}\right) \end{array}$$

where σ is conductivity, *T* is the temperature, *T*_0_ corresponds to the characteristic temperature when σ is infinite, σ_0_ is a reference viscosity, and *D* is a structural constant depend on each ionic liquid. From Figure 2, an obvious influence of the temperature on the conductivity is observed.

The alternation of conductivities of the four studied ILs in this paper is not linear correlation with the decreasing of viscosities. The higher viscosities of ILs, the slower increase of conductivities. Then becoming quicker due to continuously decrease of viscosities with the temperatures increased. This illustrates that high viscosity will hinder the diffusion and transfer rate of charge, thus lead to the low conductivity of IL. The cationic structure, including ionic size, formula weight and conjugated structure of imidazolium ring will also influence the conductivities of ILs. The conductivity of ILs can be described as Equation (3) (Rüther et al., 2013):

(3) $$\begin{array}{c} \sigma =F\sum C_{i}^{\prime }u_{i}=yFC\left(u_{c}+u_{a}\right)=\frac{yFd}{FW}\left(u_{c}+u_{a}\right) \end{array}$$

where *u*_c_, *u*_a_ are the cation and anion mobilities, *F* is the Faraday constant, *C* is the molar concentration, *y* is the degree of dissociation and 0 < y < 1, *d* is the density, and FW is the formula weight.

The Stokes-Einstein relation correlates self-diffusivity (*D*) to viscosity is depicted of the medium even in an ionic medium. The equations are following: (Equation 4)

(4) $$\begin{array}{r} D_{\text{c}}=\frac{RT}{6\pi N_{\text{A}}r_{\text{c}}\zeta_{\text{c}}\eta }\;D_{\text{a}}=\frac{RT}{6\pi N_{\text{A}}r_{\text{a}}\zeta_{\text{a}}\eta } \end{array}$$

where ξ_a_ and ξ_c_ are the anion and cation microviscosity factors, respectively. The self-diffusivity of the ions can be also associated with ion mobility: (Equation 5)

(5) $$\begin{array}{r} D_{\text{c}}\;=\;\frac{u_{\text{c}}RT}{F}\;D_{\text{a}}\;=\;\frac{u_{\text{a}}RT}{F} \end{array}$$

Based on Equations (3–5), the relationship of conductivity and viscosity can be shown in Equation (6):

(6) $$\begin{array}{r} \sigma \;=\;\frac{yF^{2}d}{6\pi N_{\text{A}}FW}\frac{\left[{\left(\zeta_{\text{c}}r_{\text{c}}\right)}^{-1}+{\left(\zeta_{\text{a}}r_{\text{a}}\right)}^{-1}\right]}{\eta } \end{array}$$

where *N*_A_ is the Avogadro's number, *r*_a_ and *r*_c_ are the anion and cation radius. The microviscosity factor ξ_c_ and ξ_a_ relates to the specific interactions between the mobile ions in the ILs, of which is governed by interionic hydrogen-bonding and Coulombic interactions.

According to Equation (6), the conductivities of the ILs can get a reasonable degree of approximation related to their viscosities (η), formula weight (FW), densities (*d*), and radii of their ions (*r*_a_ and *r*_c_). Qualitatively, the relationship between conductivity and other physical parameters in Equation (6) was verified. Besides the obvious influence of the viscosity, the effect of ion size and formula weight must be stressed.

According to the combination of the Nernst-Einstein equation for the relationship between the self-diffusivity in a liquid and its ionic conductivity, and the Stokes–Einstein equation, the relationship of the molar conductivity (Λ) and viscosity (η) can be shown in Equation (7):

(7) $$\begin{array}{r} \Lambda \eta =\;\frac{\sigma }{C}\eta \;=\;\frac{yF^{2}}{6\pi N_{A}}\left[{\left(\zeta_{\text{c}}r_{\text{c}}\right)}^{-1}+{\left(\zeta_{\text{a}}r_{\text{a}}\right)}^{-1}\right] \end{array}$$

**Figure 3:**
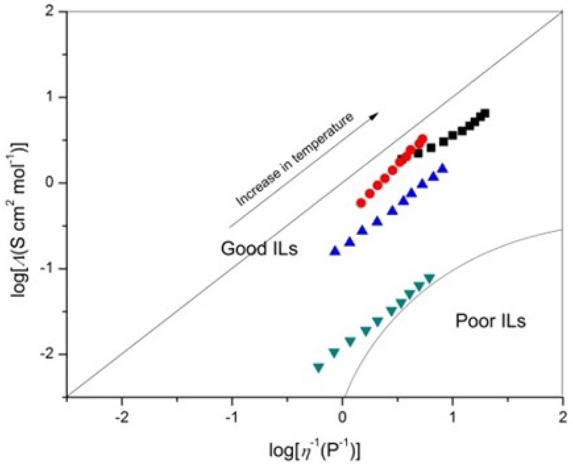
Walden plots of log(molar conductivity, Λ) against log(reciprocal viscosity η^−1^), [C_4_mim][N(CN)_2_] (black), [C_4_m_2_im][N(CN)_2_] (red), N_4442_[N(CN)_2_] (blue), and N_8444_[N(CN)_2_] (green).

The conductivity and viscosity of an IL is often combined into what is termed Walden's rule (Equation 8) (Ueno et al., 2010):

(8) $$\begin{array}{r} \Lambda \eta =\text{ constant} \end{array}$$

where Λ is the molar conductivity of the IL, and it is given by Equation (9):

(9) $$\begin{array}{r} \Lambda \;=\;\sigma M/d \end{array}$$

where *M* is the molecular weight and *d* is the density of the IL. Ideally, the Walden product (Λη) remains constant for a given IL regardless of temperature. The magnitude of the Walden product for different ILs has been shown to vary inversely with ion size. This inverse relationship between ion size and the magnitude of Λη is generally followed for the cations. Figure 3 shows the Walden plot of log(molar conductivity, Λ) against log(reciprocal viscosity η^−1^) graphs.

The magnitude of the deviation below the ideal line is presumably a result of ion aggregation, which suggests that Coulombic interactions between ions can strongly influence ionicity. Increasing cation size tends to give rise to lower conductivity, most probably due to the lower mobility of the larger cations. Moreover, the formula weight increase with the alkyl chain elongated, then increasing the van der Waals attractive forces. The bigger van der Waals forces will present an obstacle to the migration of charge, resulting in the decrease of conductivity. Although all of the DCA ILs has the same charge, the apparent Coulombic interactions are much different. Compared with quaternary ammonium cation, the synergistic effect of charge delocalization and ion size and of imidazolium cation is bigger. The π electron of the conjugated structure in imidazolium ring along with a smaller ion size gives advantage of higher mobility of the imidazolium cation and lower Coulombic interactions. A strong cation–anion attraction causes poor IL ionicity. From the deviations from the reference line in the Walden plot, [C_4_mim][N(CN)_2_], [C_4_m_2_im][N(CN)_2_] and N_4442_[N(CN)_2_] which have slight deviation can be classified as “good” ILs. Obviously, the plots of N_8444_[N(CN)_2_] have larger deviation that located at the edge of “poor” ILs as Angell et al. suggested (Xu et al., 2003) (Figure 3). In particular, the imidazolium cations exhibit clear superiority than the ammonium cations.

### Electrochemical windows (EWs)

**Figure 4:**
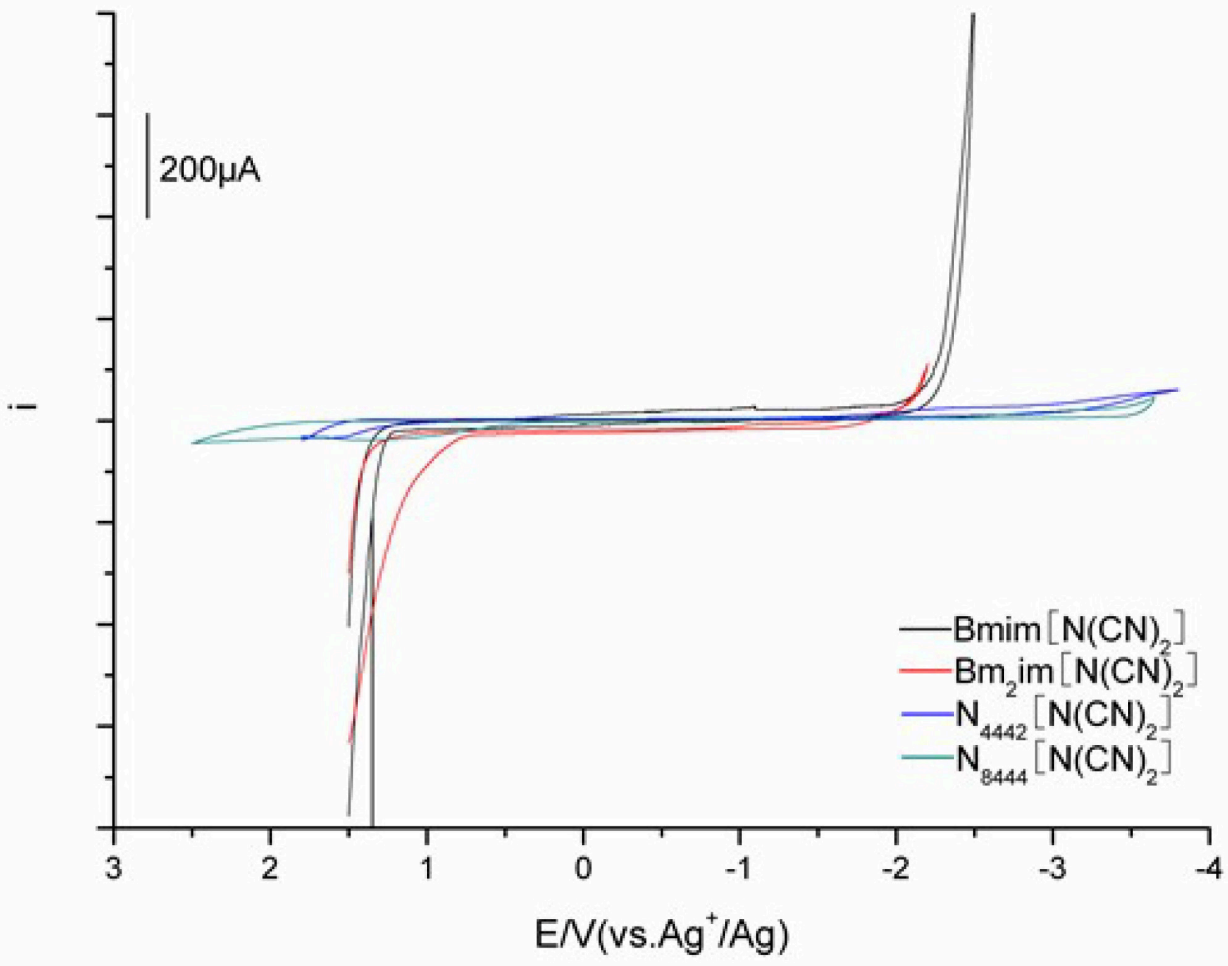
Cyclic voltammograms of [C_4_mim][N(CN)_2_] (black), [C_4_m_2_im][N(CN)_2_] (red), N_4442_[N(CN)_2_] (blue), and N_8444_[N(CN)_2_] (green) measured at 303 K.

Electrochemical behavior was performed by cyclic voltammetry using three-electrode cell with a glassy carbon (GC) rod working electrode at different temperatures. The redox potentials were recorded relative to a stable (Ag/Ag^+^) reference electrode. Figure 4 shows the representative voltammograms measured for the DCA ILs. Electrochemical window (EW, Δ*E*), associated with the electrochemical stabilities of ILs, is determined from the curve described in Figure 4. The EWs Δ*E* for [C_4_mim][N(CN)_2_] and [C_4_m_2_im][N(CN)_2_] are similar, with values of 3.63 and 3.56 V, respectively. These Δ*E* values are lower than the Δ*E* values observed for [C_4_mim][BF_4_] (4.1 V) and [C_4_mim][PF_6_] (4.2 V) (Schröder et al., 2000; Zhang et al., 2014). However, the ILs consisted of fluorine component are environmentally unfriendly because of their fluorine release. In fact, the Δ*E* value of [C_4_mim][N(CN)_2_] is higher than many ILs normally about 2–3 V (Wicelinski et al., 1987). The Δ*E* value ~4.5 V is observed for N_4442_[N(CN)_2_] (4.47 V). A further increase in the Δ*E* is found for N_8444_[N(CN)_2_], with the value up to 4.80 V. The data clearly indicates N_8444_[N(CN)_2_] exhibits the highest electrochemical stability among the four DCA ILs. The ${\text{N}}_{8444}^{+}$ cation provides better protection against electrochemical oxidation and reduction. The Δ*E* values of the DCA ILs are relatively wide, which give potential feasibility as electrolytes in some electrochemical applications.

**Figure 5:**
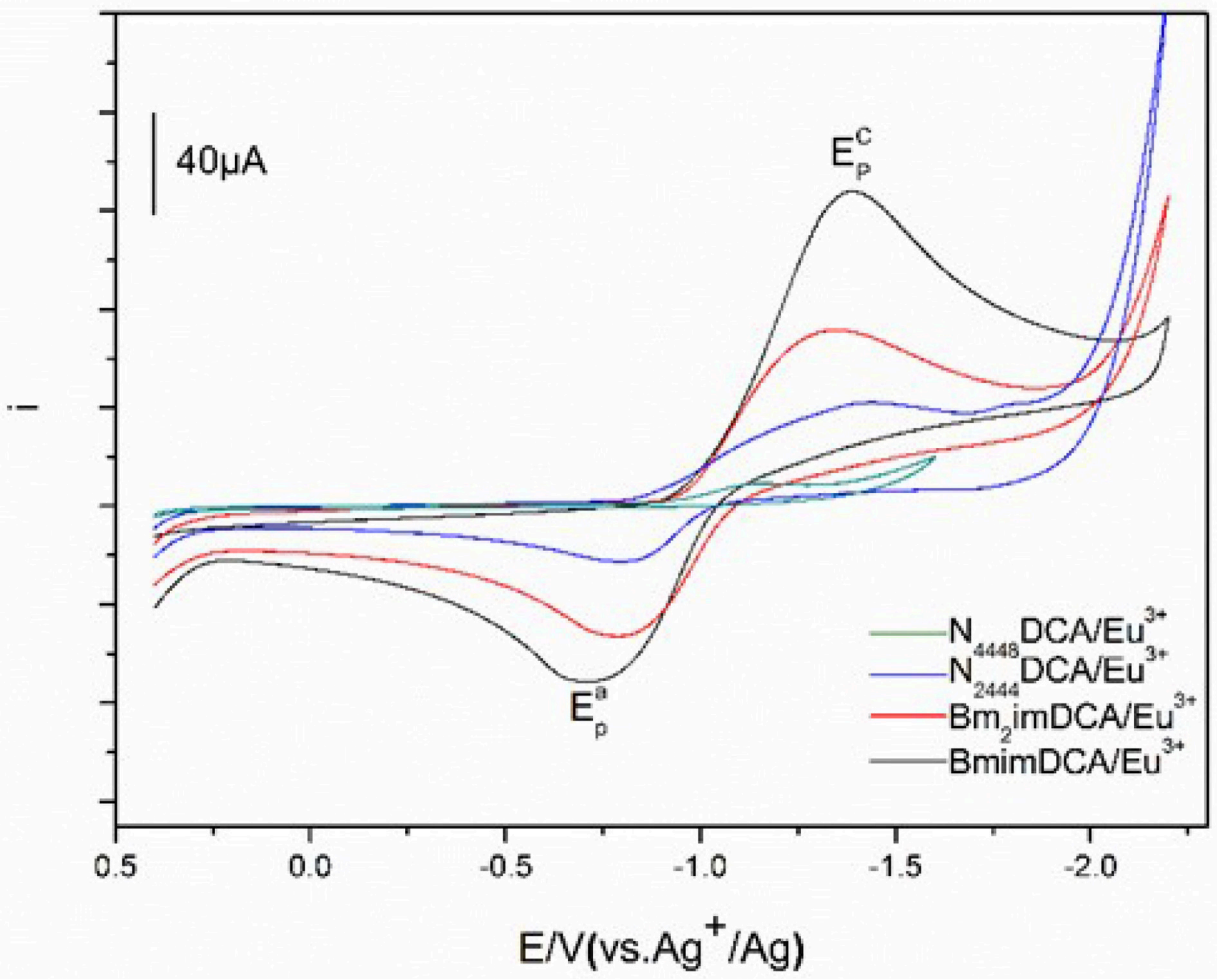
Cyclic voltammograms of Eu(III) measured in [C_4_mim][N(CN)_2_] (black), [C_4_m_2_im][N(CN)_2_] (red), N_4442_[N(CN)_2_] (blue), and N_8444_[N(CN)_2_] (green) at 303 K.

Cyclic voltammetry of Eu(III) in DCA ILs. The cyclic voltammograms of a solution of 50 mM Eu(III) in these ILs recorded at GC electrode are shown in Figure 5. The cyclic voltammograms of Eu(III) in [C_4_mim][N(CN)_2_], [C_4_m_2_im][N(CN)_2_], N_4442_[N(CN)_2_], N_8444_[N(CN)_2_] consists of quasi-reversible waves. At 303 K, a cathodic peak and an anode peak potentials of Eu(III) in [C_4_mim][N(CN)_2_], are observed around −1.39 V and −0.71 V owing to the reduction and oxidation of Eu(III). What's more, there is no deposition of elemental europium during the potentiostatic reduction. Therefore, the reduced product in this system is probably the divalent europium complex, Eu(II). The cyclic voltammetry analysis of the Eu(III)/Eu(II) in [C_4_mim][N(CN)_2_] is a quasi-reversible. Similar cyclic voltammograms determined in other three DCA ILs were also assembled, with an anode peak potentials of −0.79 V ([C_4_m_2_im][N(CN)_2_]), −0.64 V (N_4442_[N(CN)_2_]) and −0.59 V (N_8444_[N(CN)_2_]), respectively.

**Figure 6:**
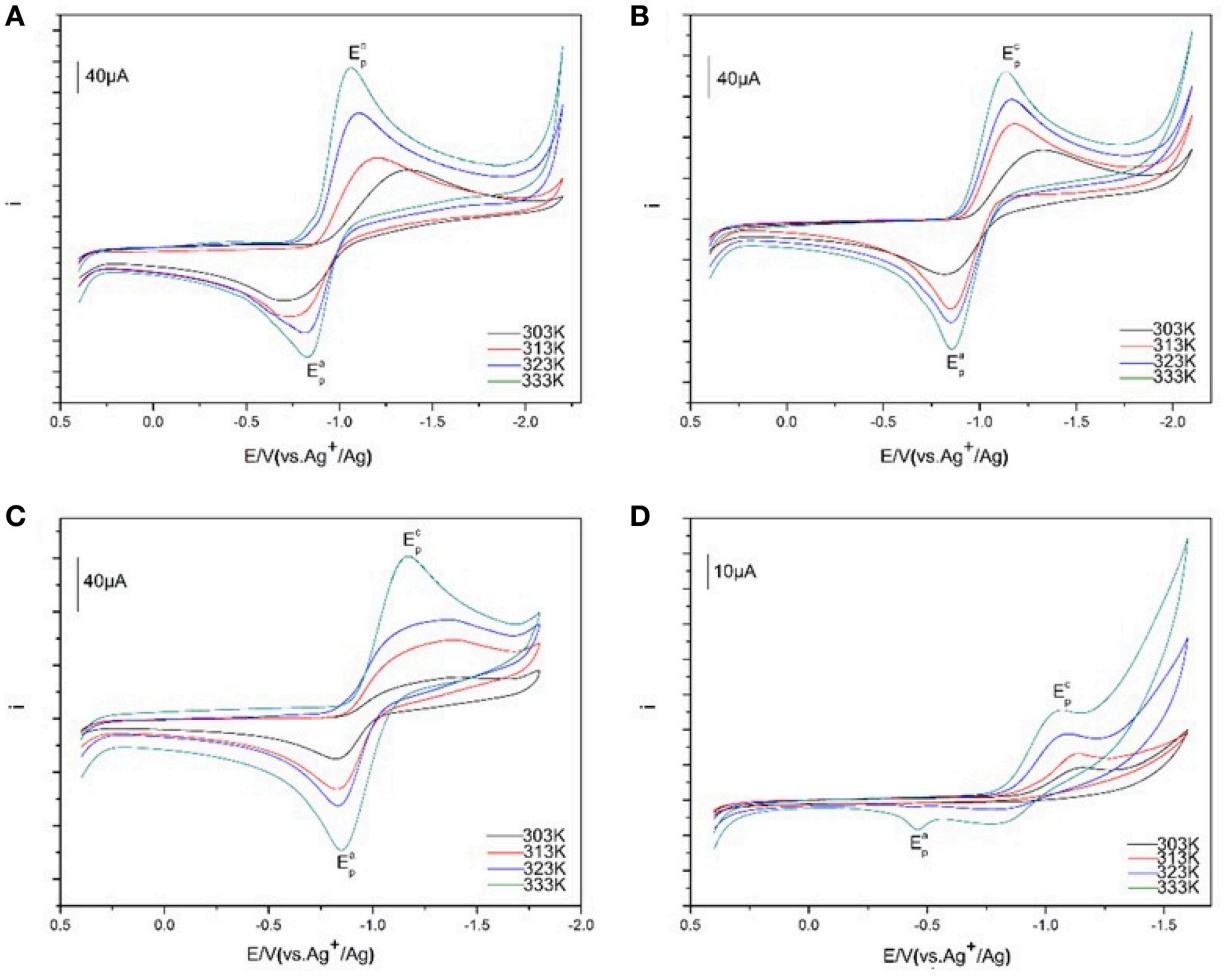
Cyclic voltammograms of Eu(III) in [C_4_mim][N(CN)_2_] **(A)**, [C_4_m_2_im][N(CN)_2_] **(B)**, N_4442_[N(CN)_2_] **(C)**, and N_8444_[N(CN)_2_] **(D)** with Ag/AgCl as reference electrode at the scan rate of 100 mV/s from 303 to 333 K.

The cyclic voltammograms of 50 mM Eu(III) at various temperatures were assembled in Figure 6. The temperatures were controlled in accuracy and selected as 303, 313, 323, and 333 K, respectively. An increase of the current intensities for Eu(III) is found in [C_4_mim][N(CN)_2_] along with the rise of temperature. A cathodic peak potentials of Eu(III) are significantly higher than the values observed at lower temperature. While, a slight reduce is recorded for an anode peak potential at higher temperature. For example, at 303 K the cathodic peak potential for Eu(III) is −1.42 V whereas the value is significantly less negtive, reaching ~ −1.02 V. The tendency of Eu(III)/Eu(II) redox reaction shows more quasi-reversible with the increase of temperature. A similar tendency is also found in [C_4_m_2_im][N(CN)_2_], N_4442_[N(CN)_2_], and N_8444_[N(CN)_2_]. This feature is associated with the mass transition caused by the viscosity and conductivity of the solvent, which depends on temperature closely (Nockemann et al., 2006). For these ILs, their viscosities decreased and the conductivities increased with the increasement of temperature, which are contributed to the diffusion of trivalent lanthanide ion Eu(III). Thus, many parameters related to transport properties are also temperature-dependent for the variation of the viscosity of ILs, such as conductivity, diffusion coefficient, and charge transfer rate. The diffusion rate became faster as the temperature increased.

**Figure 7:**
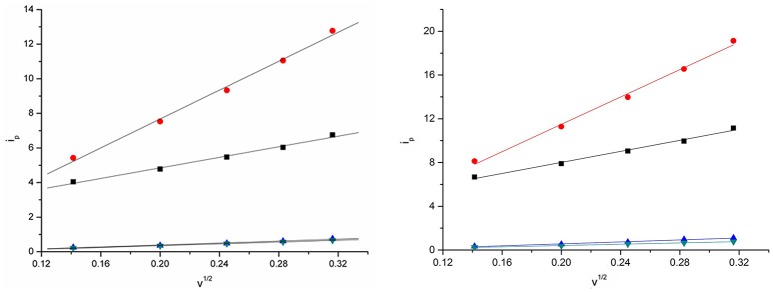
Cathodic peak current intensities as a function of ν^1/2^ of Eu(III) measured in [C_4_mim][N(CN)_2_] (black), [C_4_m_2_im][N(CN)_2_] (red), N_4442_[N(CN)_2_] (blue), and N_8444_[N(CN)_2_] (green) at 303 K **(left)** and 333 K **(right)**.

The relation between the current intensities of cathodic peak (*i*_p_) and the square-root of the potential scan rate (ν^1/2^) is shown in Figure 7 at 303 and 333 K. The plots show that there exist a positive correlation between the cathodic peak current intensity and the square-root of the potential scan rate. Good linear relationships were obtained for Eu(III) in the four DCA ILs. In addition, the value of the cathodic current densities increased as the temperature raised, which might be generated from the diffusion coefficients and charge transfer rates of the Eu(III) in DCA ILs. These results indicate that the electrode reaction kinetics is controlled by the mass transport under semi-infinitive linear diffusion conditions.

**Figure 8:**
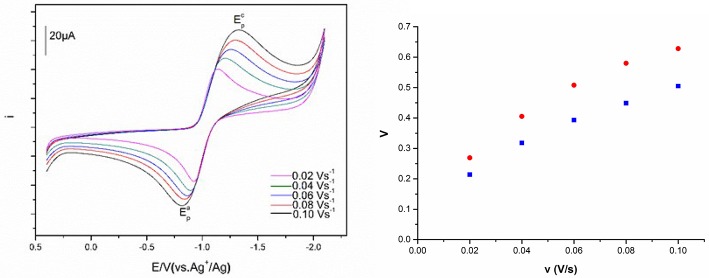
Cyclic voltammograms of Eu(III) measured in [C_4_m_2_im][N(CN)_2_] and the peak potential separation values of [C_4_m_2_im][N(CN)_2_] (blue) and N_8444_[N(CN)_2_] (red) at different scan rates.

Figure 8 described the cyclic voltammograms and the peak potential separation values of Eu(III) at various scan rates. The redox peak potentials of Eu(III) in these ILs at various scan rates are obviously observed. Along with the scan rate change, both current intensity and peak potential are moved. The Eu(III)/Eu(II) redox reaction shows more reversible at the low scan rate, and the separation of the cathodic and anodic peak potentials would be shift to cathode and anode, respectively, as the scan rate raised.

Diffusion coefficients and energy of activation (*E*_a_) of Eu(III) in DCA ILs. The diffusion coefficients of Eu(III) in DCA ILs were measured by series of electrochemical analyses. The relationship between cathodic peak current and diffusion coefficient (*D*_o_) for a quasi-reversible system can be predicted as Equation (10) (Bard and Faulkner, 1980; Molina et al., 2013):

(10) $$\begin{array}{c} i_{p}=0.496nFAC_{o}^{*}D_{o}^{1/2}v^{1/2}{\left(\frac{\alpha n_{\alpha }F}{RT}\right)}^{1/2} \end{array}$$

where *A* is the electrode area in cm^2^, $C_{\text{o}}^{*}$ is the Eu(III) concentration in mmol·L^−1^, *D*_o_ is the diffusion coefficient in cm^2^·s^−1^, ν is the potential scan rate in V·s^−1^, *F* is the Faraday constant, α is the charge transfer coefficient, *n* is the number of electron transfer, *n*α is the number of electron transfer in the rate determining step, and *T* is the absolute temperature in K. The value of α*n*_α_ can be determined using Equation (11) (Matsumiya et al., 2008):

(11) $$\begin{array}{r} \left|E_{p}^{\text{c}}-E_{p/2}^{\text{c}}\right|\;=\;\frac{1.857RT}{\alpha n_{\alpha }F} \end{array}$$

where $E_{\text{p}}^{\text{c}}$ is the cathodic potentials, $E_{\text{p}/2}^{\text{c}}$ is the half wave potentials, and |$E_{\text{p}}^{\text{c}}$-$E_{\text{p}/2}^{\text{c}}$| is the absolute value of the difference between $E_{\text{p}}^{\text{c}}$ and $E_{\text{p}/2}^{\text{c}}$. The data of cathodic peak ($E_{\text{p}}^{\text{c}}$), anodic peak ($E_{\text{p}}^{\text{a}}$) potentials and the average of cathodic and anodic peak potential, ($E_{\text{p}}^{\text{c}}\;+E_{\text{p}}^{\text{a}}$)/2, at various temperatures are summarized in Table 2.

**Table 2:** Peak potentials ($E_{\text{p}}^{\text{c}}$ and $E_{\text{p}}^{\text{a}}$), average of cathodic and anodic peak potentials [($E_{\text{p}}^{\text{c}}\;+E_{\text{p}}^{\text{a}}$)/2], diffusion coefficients (*D*_o_), electron transfer rate constant (*k*_s_), and energy of activation (*E*_a_) of Eu(III) measured in the DCA ILs at different temperatures.

**Ionic liquid**	**T (K)**	**$E_{\text{p}}^{\text{c}}$ (V)**	**$E_{\text{p}}^{\text{a}}$ (V)**	**($E_{\text{p}}^{\text{c}}$ + $E_{\text{p}}^{\text{a}}$)/2 (V)**	***D*_o_ × 10^8^ (cm^2^·s^−1^)**	***E*_a_ (kJ·mol^−1^)**	***k*_s_ × 10^4^ (cm·s^−1^)**
[C_4_mim][N(CN)_2_]	303	−1.349	−0.710	−1.030	26.5	27.30	4.24
	313	−1.235	−0.737	−0.986	34.6		4.72
	323	−1.106	−0.816	−0.961	52.6		5.30
	333	−1.063	−0.829	−0.946	67.9		6.09
[C_4_m_2_im][N(CN)_2_]	303	−1.327	−0.791	−1.059	6.52	31.11	2.12
	313	−1.179	−0.865	−1.022	9.19		2.25
	323	−1.157	−0.869	−1.013	13.72		2.92
	333	−1.113	−0.874	−0.994	19.53		3.41
N_4442_[N(CN)_2_]	303	−1.232	−0.636	−0.934	0.0744	41.06	0.108
	313	−1.207	−0.738	−0.973	0.1076		0.163
	323	−1.196	−0.866	−1.031	0.1647		0.214
	333	−1.189	−0.912	−1.051	0.3304		0.362
N_8444_[N(CN)_2_]	303	−1.213	−0.585	−0.899	0.0615	34.88	0.102
	313	−1.181	−0.625	−0.903	0.0799		0.119
	323	−1.170	−0.669	−0.919	0.1267		0.130
	333	−1.162	−0.698	−0.930	0.2115		0.145

From the Equations (10, 11), the diffusion coefficients of Eu(III) in these ILs can be determined, the values are shown in Table 2. The diffusion coefficients can be regarded as a function of *T*. Changes of temperature gives an increase or decrease of the diffusion coefficients and the charge transfer coefficients. The diffusion coefficient of Eu(III) in [C_4_mim][N(CN)_2_] is about 26.5 × 10^−8^ cm^2^·s^−1^ at 303 K, and as high as 67.9 × 10^−8^ cm^2^·s^−1^ at higher temperature. Low viscosity and high conductivity at high temperature result in more efficient mass transport with high value of diffusion coefficient. The viscosity and conductivity of IL are of importance to influent the application of IL as a solvent because that they will influence the transport properties of IL for some metal ions, including diffusion coefficient and charge transfer rate etc. Similar trends are also found for Eu(III) in other DCA ILs. The magnitude of diffusion coefficients for Eu(III) in [C_4_mim][N(CN)_2_] and [C_4_m_2_im][N(CN)_2_] are around ~10^−7^ cm^2^·s^−1^, while the values for Eu(III) recorded in N_4442_[N(CN)_2_] and N_8444_[N(CN)_2_] are ~10^−10^ cm^2^·s^−1^ at 303 K, then being ~10^−9^ cm^2^·s^−1^ at higher temperature. Such values performed in the imidazolium DCA ILs are 10^2^–10^3^ times larger than those of Eu(III) in N_4442_[N(CN)_2_] and N_8444_[N(CN)_2_], which are attributed to their higher viscosity and lower conductivity of the quaternary ammonium-based ILs relative to the imidazolium-based ILs. A reduce in the transport properties of quaternary ammonium DCA ILs hinders the diffusion of Eu(III) in ILs. This phenomenon shows that the electrostatic interaction around Eu(III) in the quaternary ammonium DCA ILs may be weaker than those of Eu(III) in the imidazolium-based ILs.

**Figure 9:**
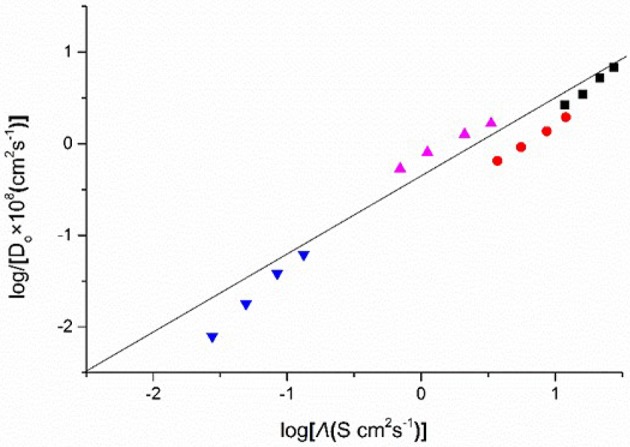
Logarithm of diffusion coefficients (*D*_o_) of Eu(III), against as a function of log(molar conductivity, Λ), measured in [C_4_mim][N(CN)_2_] (black), [C_4_m_2_im][N(CN)_2_] (red), N_4442_[N(CN)_2_] (blue), and N_8444_[N(CN)_2_] (violet).

Figure 9 shows the plots of the diffusion coefficients (log*D*_o_) of Eu(III) in four DCA ILs and the molar conductivities (logΛ) of these ILs. A linear relationship is observed, which indicates that the diffusion coefficients are correlative with molar conductivity. Although the mechanism how the friction on the translational motion affects the relaxation of the ionic atmosphere around the Eu ion is still unknown, based on the plots, the diffusion coefficients may be predicted as a function of molar conductivity. Thus, for ILs, molar conductivity is a valuable quantity to construct linear relationship with the transport properties. The calculated data of diffusion coefficients may not be accurate and can be used as reference data.

**Figure 10:**
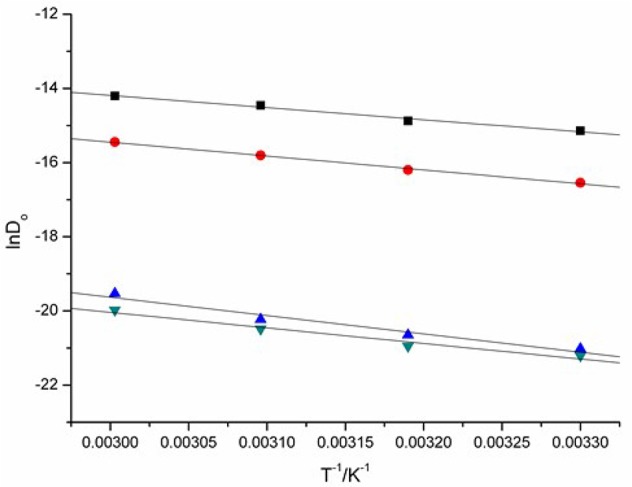
ln*D*_o_ of Eu(III), as a function of *T*^−1^ measured in [C_4_mim][N(CN)_2_] (black), [C_4_m_2_im][N(CN)_2_] (red), N_4442_[N(CN)_2_] (blue), and N_8444_[N(CN)_2_] (green).

The energy of activation (*E*_a_) of the reduction of Eu(III) to Eu(II) can be determined from the slope of ln*D*_o_ against 1/*T* (Figure 10) and the data were also given in Table 2. The raise of the *E*_a_ magnitude is parallel to the increase of the conductivities of these ILs. The reduction of Eu(III) to Eu(II) exhibits the values of *E*_a_ DCA ILs around 27.30 to 41.06 kJ·mol^−1^.

Charge transfer rate constants (*k*_s_) of Eu(III) in ILs. The major factors that affect the reduction process of Eu(III) to Eu(II) in DCA ILs relate to both diffusion and charge transfer kinetics. For a quasi-reversible system, the charge transfer rate constant (*k*_s_), depended on both diffusion coefficient and transfer coefficient, can be described as Equation (12) (Brown and Sandifer, 1986; Rao et al., 2010):

(12) $$\begin{array}{r} k_{s}\;=\;2.18{\left[\frac{D_{\text{o}}\left(\alpha n_{\alpha }\right)vF}{RT}\right]}^{1/2}exp\left[\frac{\alpha^{2}nF\left(E_{\text{p}}^{\text{c}}-E_{\text{p}}^{\text{a}}\right)}{RT}\right] \end{array}$$

The data of charge transfer rate constants (*k*_s_), the cathodic and anodic peak potentials of Eu(III) are summarized in Table 2. The charge transfer rate constants of Eu(III) in [C_4_mim][N(CN)_2_] and [C_4_m_2_im][N(CN)_2_] are higher than the values recorded in N_4442_[N(CN)_2_] and N_8444_[N(CN)_2_]. The magnitude of charge transfer rate constants of Eu(III) in [C_4_mim][N(CN)_2_] and [C_4_m_2_im][N(CN)_2_] are observed to be the order of 10^−4^ cm·s^−1^, while those determined in N_4442_[N(CN)_2_] and N_8444_[N(CN)_2_] are of the order of ~10^−5^ cm·s^−1^. Such data increased when the temperature increased. The lower viscosity and higher conductivity of ILs at higher temperatures may facilitate electron transfer at electrode-electrolyte interphase. Thus, an increase of the *k*_s_ for Eu(III) in BmimBr can be found at higher temperature. The electrode reaction can be classified as, reversible when *k*_s_ ≥ 0.3*v*^1/2^ cm·s^−1^, quasi-reversible when 0.3*v*^1/2^ ≥ *k*_s_ ≥2 × 10^−5^*v*^1/2^ cm·s^−1^, and irreversible when *k*_s_ ≤ 2 × 10^−5^*v*^1/2^ cm·s^−1^. Based on the *k*_s_ values determined using Equation (12), the electrode reactions of Eu(III) to Eu(II) in DCA ILs are confirmed as quasi-reversible reactions.

Determination of Gibbs energy change of Eu(III) in ILs. Gibbs energy, Δ*G*, is of central importance to reaction. The reductions of Eu(III) to Eu(II) in [C_4_mim][N(CN)_2_] and [C_4_m_2_im][N(CN)_2_] are just expressed as:

(13) $$\begin{array}{r} 2\text{EuC}{\text{l}}_{3}\;↔\;2\text{EuC}{\text{l}}_{2}\;+\text{ C}{\text{l}}_{2} \end{array}$$

The apparent standard potential, $E_{Eu\left(III\right)/Eu\left(II\right)}^{0^{*}}$, is related to the cathodic and anodic peak potentials and their relation are given:

(14) $$\begin{array}{r} E_{p}^{a}\;=\;E_{Eu\left(III\right)/Eu\left(II\right)}^{0^{*}}+1.11\frac{RT}{nF}-\frac{RT}{nF}ln\left(\frac{\sqrt{D_{Eu\left(III\right)}}}{\sqrt{D_{Eu\left(II\right)}}}\right) \end{array}$$

(15) $$\begin{array}{r} E_{p}^{c}\;=\;E_{Eu\left(III\right)/Eu\left(II\right)}^{0^{*}}-1.11\frac{RT}{nF}-\frac{RT}{nF}ln\left(\frac{\sqrt{D_{Eu\left(III\right)}}}{\sqrt{D_{Eu\left(II\right)}}}\right) \end{array}$$

Because the number of electrons transfer (*n*) in the reduction of Eu(III) equals to 1, the expression of $E_{Eu\left(III\right)/Eu\left(II\right)}^{0^{*}}$, a function of temperature, can be predicted as Equations (14, 15).

(16) $$\begin{array}{r} E_{Eu\left(III\right)/Eu\left(II\right)}^{0^{*}}\;=\;\frac{E_{p}^{c}+E_{p}^{a}}{2}+\frac{RT}{F}ln\left(\frac{\sqrt{D_{Eu\left(III\right)}}}{\sqrt{D_{Eu\left(II\right)}}}\right) \end{array}$$

**Figure 11:**
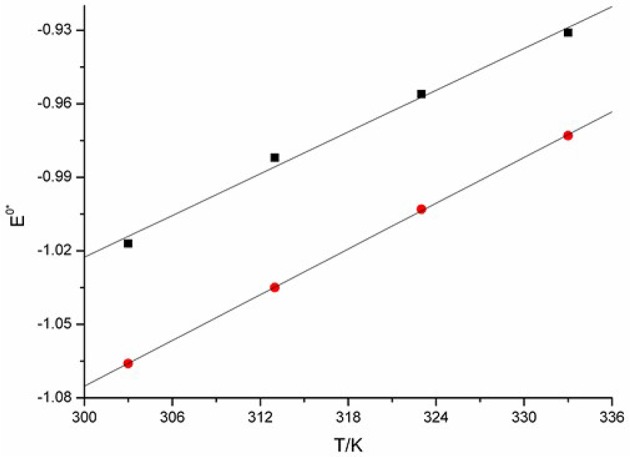
Plots of ${\text{E}}_{\text{Eu}\left(\text{III}\right)/\text{Eu}\left(\text{II}\right)}^{0*}$ against *T* measured in [C_4_mim][N(CN)_2_] (black), and [C_4_m_2_im][N(CN)_2_] (red).

The relation between $E_{Eu\left(III\right)/Eu\left(II\right)}^{0^{*}}$ and temperature can be obtained from linear regression of the experimental data. The plots were shown in Figure 11. The alternation of $E_{Eu\left(III\right)/Eu\left(II\right)}^{0^{*}}$ are linear correlation with the increasing of *T*. Thus, the apparent standard potential $E_{Eu\left(III\right)/Eu\left(II\right)}^{0^{*}}$ can be further simplified and expressed as Equations (17, 18).

(17) $$\begin{array}{r} E_{Eu\left(III\right)/Eu\left(II\right)}^{0^{*}}\;=\;-1.88+2.84\times 10^{-3}T\;\left(\text{K}\right)\text{ vs}.\;\left(\text{C}{\text{l}}_{2}/\text{C}{\text{l}}^{-}\right) \end{array}$$

(18) $$\begin{array}{r} E_{Eu\left(III\right)/Eu\left(II\right)}^{0^{*}}\;=\;-2.01+3.11\times 10^{-3}T\;\left(\text{K}\right)\text{ vs}.\;\left(\text{C}{\text{l}}_{2}/\text{C}{\text{l}}^{-}\right) \end{array}$$

From the expression we see, the $E_{Eu\left(III\right)/Eu\left(II\right)}^{0^{*}}$ can be recognized as a function of *T*. In this work, a linear correlation of $E_{Eu\left(III\right)/Eu\left(II\right)}^{0^{*}}$ with temperature is found.

Assuming the solutions of Eu(III) in the DCA ILs are dilute and the activity coefficient are negligible, the standard Gibbs energy of the reaction EuCl_2_ + 1/2Cl_2_ → EuCl_3_ can be estimated using the expression (19).

(19) $$\begin{array}{r} \Delta G^{0}\;\left(\text{EuC}{\text{l}}_{3}\right)\;=\;-nFE_{Eu\left(II\right)/Eu\left(III\right)}^{0^{*}} \end{array}$$

where $E_{Eu\left(II\right)/Eu\left(III\right)}^{0^{*}}$ is the apparent standard potential of oxidation of Eu(II) to Eu(III). The relationships of the standard Gibbs energy of the reaction in [C_4_mim][N(CN)_2_] and [C_4_m_2_im][N(CN)_2_] and *T* just as given below expressions (20, 21), respectively.

Based on Equations (17–19), the standard Gibbs energy expression is to be a linear function of temperature.

(20) $$\begin{array}{r} \Delta G_{EuCl_{3}}^{0}\;\left(\text{kJ mo}{\text{l}}^{-1}\right)=-181.42+0.2741\;T\;\left(\text{K}\right) \end{array}$$

(21) $$\begin{array}{r} \Delta G_{EuCl_{3}}^{0}\;\left(\text{kJ mo}{\text{l}}^{-1}\right)=-193.96+0.3001\;T\;\left(\text{K}\right) \end{array}$$

The expressions of Equations (20, 21) show that the standard entropy (Δ$S_{\text{EuCl}3}^{0}$) of the reaction EuCl_2_ + 1/2 Cl_2_ → EuCl_3_. According to the expression Δ*G* = Δ*H* – *T*Δ*S*, the values of Δ$S_{\text{EuCl}3}^{0}$ are found to be negative around −0.3. The result shows that there is a decrease entropy during the reaction process, accompanied by the generation of more ordered EuCl_3_ from less ordered substrates. The results indicate that the entropy of reaction decrease, which are accord with the stoichiometric number of substances of the reaction reduced.

## Conclusions

Four DCA ILs, [C_4_mim][N(CN)_2_], [C_4_m_2_im][N(CN)_2_], N_4442_[N(CN)_2_], and N_8444_[N(CN)_2_], were prepared and characterized. Except for [C_4_m_2_im][N(CN)_2_] with a melting point 299 K, other DCA ILs are all room temperature ILs with good thermal stability. Their transport properties including viscosities, conductivities, and electrochemical properties, were studied in detail at different temperatures. The influence factors on the viscosity and ionic conductivity of these ILs have been discussed. A decrease of the viscosity and increase of ionic conductivity of these ILs are recorded as the temperature increase. Besides temperature, hydrogen-bond, van der Waals force, entropy and charge distribution of cations are all possible affecting factors on the viscosity. Although the effect of viscosity on the conductivity is very significant, the cationic structure, including ionic size, formula weight, and conjugated structure of imidazolium ring could not be ignored. Based on the Walden plots, the cation–anion attraction among IL could be estimated. [C_4_mim][N(CN)_2_], [C_4_m_2_im][N(CN)_2_], and N_4442_[N(CN)_2_] can be classified as “good” ILs. While N_8444_[N(CN)_2_] locates at the edge of “poor” ILs. So in order to design low viscous and high conductive ILs, the importance of the cationic structure must be kept in mind.

A series of electrochemical analyses of the DCA ILs have been performed. These ILs give relatively high values of EWs, with the order of N_8444_[N(CN)_2_] > N_4442_[N(CN)_2_] > [C_4_mim][N(CN)_2_] ≈ [C_4_m_2_im][N(CN)_2_]. Such feature indicates that the DCA ILs are potential candidates for electrolytes in electrochemical applications. Meanwhile, the electrochemical behaviors of Eu(III) in these DCA ILs at GC working electrode at various temperatures 303–333 K were found. A series of quasi-reversible waves of Eu(III) were recorded by cyclic voltammetry. The electrochemical properties of the DCA ILs are also dominated by the cationic structures. The current intensity (*i*_p_), the diffusion coefficients (*D*_o_), the charge transfer rate constants (*k*_s_) of Eu(III) in DCA ILs all increased with the molar conductivities increased. Moreover, the apparent standard potentials [${\text{E}}_{\text{Eu}\left(\text{III}\right)/\text{Eu}\left(\text{II}\right)}^{0^{*}}$] and the standard Gibbs energy of the reduction of Eu(III) to Eu(II) were also determined.

In summary, the effect of the cationic structures including ionic size, formula weight and conjugated structure of imidazolium ring on the transport properties is very significant. The structure-property relationships of DCA ILs will be very useful to help us to understand other IL families and design novel functionalized ILs fulfilling specific demand.

## Author contributions

LH and G-HT: designed the research; W-LY, XY, and SQ: prepared the samples and did determinations; W-LY, XY, YX, and G-HT: were involved in the data analysis; W-LY, XY, LH, and G-HT: wrote the manuscript.

### Conflict of interest statement

The authors declare that the research was conducted in the absence of any commercial or financial relationships that could be construed as a potential conflict of interest.
